# Dimethyl α-ketoglutarate ameliorates cisplatin-induced acute kidney injury by modulating mitophagy through the PINK1/Parkin pathway

**DOI:** 10.1186/s40001-025-03010-7

**Published:** 2025-08-13

**Authors:** Haijing Dou, Huihui Hao, Ruoyi Zhao, Hailun Li, Fangshu Wei, Yong Xu, Donghui Zheng, Juan Xie, Xiang Li

**Affiliations:** 1 Department of Clinical Laboratory, The Affiliated Huai’an Hospital of Xuzhou Medical University and Huai’an Second People’s Hospital, Huai’an, 223002 China; 2 Department of Pharmacology, Jiangsu College of Nursing, Huai’an, 223002 Jiangsu China; 3 Department of Nephrology, The Affiliated Huai’an Hospital of Xuzhou Medical University and Huai’an Second People’s Hospital, Huai’an, 223002 China; 4 Department of Nephrology, The Affiliated Huaian No. 1 People’s Hospital of Nanjing Medical University, Huai’an, 223300 China

**Keywords:** Alpha-ketoglutarate, Mitophagy, Cisplatin, Acute kidney injury, Network pharmacology

## Abstract

**Background:**

Mitochondrial dysfunction and abnormal energy metabolism are key determinants of the progression of acute kidney injury (AKI). α-Ketoglutarate (AKG) is an intermediate metabolite of the tricarboxylic acid cycle and plays a crucial role in energy metabolism and amino acid synthesis. However, the role of AKG in AKI therapy remains incompletely understood.

**Methods:**

Cisplatin (CIS) was employed to establish acute kidney injury models in mice and cells, with DM-AKG administered as an intervention. Network pharmacology was utilized to predict the target genes and pathway enrichment of AKG in the treatment of AKI. Apoptosis was assessed using flow cytometry, and cell viability was determined via the CCK-8 assay. The levels of intracellular reactive oxygen species (ROS) and mitochondrial membrane potential (MMP) were assessed using optical microscopy. Renal function was evaluated using absorbance spectroscopy. Hematoxylin–eosin (H&E) staining was used to examine the pathological changes in renal tissues across different groups. The ultrastructure of mitochondria was examined using transmission electron microscopy. Protein expression levels of KIM-1, Caspase-3, DRP1, MFN1, PINK1, and Parkin were evaluated using Western blot analysis. The expression of PINK1 and Parkin was examined by immunohistochemistry.

**Results:**

Herein, we demonstrate that dimethyl α-ketoglutarate (DM-AKG), an AKG derivative with favorable cell membrane permeability, effectively ameliorates cisplatin (CIS)-induced AKI. Further network pharmacological analyses revealed that AKG could treat AKI through 91 potential targets of action. Moreover, Gene Ontology (GO) and Kyoto Encyclopedia of Genes and Genomes (KEGG) pathway analyses showed significant enrichment of pathways related to mitochondria and energy metabolism. Furthermore, in a CIS-treated HK-2 cell model, we found that exogenous DM-AKG supplementation improved mitochondrial dynamics (increased expression of the mitochondrial fusion protein MFN1 and decreased expression of the mitochondrial fission protein DRP1), increased mitochondrial membrane potential, and decreased reactive oxygen species generation. Consistent with these findings, in the CIS-AKI mouse model, DM-AKG similarly improved mitochondrial morphology, structure, and dynamics, as well as increased mitophagy observed by electron microscopy.

**Conclusion:**

These results suggest that DM-AKG may exert a therapeutic effect on AKI by improving mitochondrial function. Regarding the molecular mechanism, we confirmed that DM-AKG could increase mitophagy and promote the clearance of damaged mitochondria by activating the PINK1/Parkin pathway, which could play a protective role in the kidney. In conclusion, our study provides a novel strategy for the effective treatment of AKI.

## Introduction

Acute kidney injury (AKI) is a common clinical syndrome characterized by a rapid decline in kidney function and is caused by a variety of factors [1]. AKI has emerged as a significant public health concern, affecting millions of patients worldwide and leading to decreased survival rates and accelerated progression of chronic kidney disease (CKD) [2, 3].

The kidney is a “high-energy-consuming” organ, with an extremely high demand for ATP during the execution of physiological functions such as nutrient reabsorption, acid–base and electrolyte balance, and hemodynamics [4]. Under physiological conditions, the tricarboxylic acid cycle/oxidative phosphorylation is the most efficient pathway for ATP production in the kidney [5, 6]. Accumulating evidence indicates that mitochondrial dysfunction and alterations in cellular energy metabolism play a crucial role in the development and progression of AKI, emerging as a key breakthrough direction for achieving precise and effective treatment of AKI [7, 8].

α-Ketoglutarate (AKG) is an endogenous intermediate metabolite in the tricarboxylic acid cycle and plays a crucial role in various energy metabolism processes, including amino acids, glucose, and fatty acids [9, 10]. Studies have reported that α-ketoglutarate (AKG) ameliorates stress-overload-induced cardiac dysfunction in mice via NAD-SIRT1 signal-mediated mitophagy and ferroptosis [11]. Additionally, AKG prevents high-fat diet-induced mitochondrial dysfunction and oxidative stress in fatty liver by activating the AMPK-PGC-1α/Nrf2 pathway [11, 12]. Furthermore, AKG alleviates high-fat diet-induced endothelial injury by inhibiting oxidative stress and mitochondrial dysfunction via the ERK-NRF2 signaling pathway [13]. Dimethyl α-ketoglutarate (DM-AKG) is a derivative of AKG with two additional methyl groups attached to its molecular structure. This modification significantly enhances its ability to penetrate the cell membrane, thereby increasing intracellular AKG levels [14]. However, to date, the specific effects and underlying molecular mechanisms of DM-AKG in AKI have not been fully elucidated.

In this study, we will investigate the therapeutic efficacy of DM-AKG in CIS-induced AKI (CIS-AKI) using both in vitro cell models and in vivo models. Subsequently, we will employ network pharmacology techniques to identify the core targets involved in the treatment of AKI by DM-AKG and to deeply explore the closely related gene-enriched pathways. Finally, we will further explore the molecular mechanisms underlying DM-AKG’s treatment of AKI using relevant experimental methods. In summary, our research findings are expected to provide valuable experimental evidence for the effective prevention and treatment of AKI.

## Materials and methods

### Experimental validation study

#### Antibody reagents

The primary antibodies used in this study are as follows: Parkin (ES3151, ELK Biotechnology), PINK1 (ES10983, ELK Biotechnology), DRP1 (ES2202, ELK Biotechnology), Caspase-3 (ES1854, ELK Biotechnology), MFN1 (ES14955, ELK Biotechnology), and KIM-1 (ES8662, ELK Biotechnology). GAPDH (D110016, Sangon Biotech) was used as a loading control. The secondary antibody was a horseradish peroxidase-conjugated goat anti-rabbit antibody (BS13278, Bioworld).

### Cell culture and treatment

Human renal tubular epithelial cells (HK-2 cells) (Servicebio Technology, Wuhan, China) were cultured in DMEM/F12 (1:1) medium supplemented with 10% heat-inactivated fetal bovine serum, penicillin (100 U/ml), and streptomycin (100 U/ml). The cellular model of cisplatin (CIS)-induced acute kidney injury (AKI) was established as previously described [15]. Briefly, when HK-2 cells reached 70–80% confluence, they were treated with a dose of 6 μg/mL cisplatin (Haosen, Jiangsu, China) and then incubated at 37 °C with 5% CO_2_ for 24 h. For DM-AKG treatment, HK-2 cells were pretreated with different doses of DM-AKG (Sigma-Aldrich, Germany) for 4 h prior to cisplatin exposure.

### Animal model and treatment

All male C57BL/6 J wild-type (WT) mice used in the study were purchased from Jiangsu Jicui Pharmachem Biotechnology Co., Ltd. The mice were randomly divided into three groups: control group (*n* = 6), CIS-AKI group (*n* = 6), and CIS-AKI + DM-AKG group (*n* = 6). The mouse model of CIS-induced AKI was established as previously described [16]. In short, AKI is administered by intraperitoneal injection of cisplatin (24 mg/kg) diluted with normal saline. The control group was injected with equal volume of normal saline. In addition, we improved the treatment of DM-AKG by intraperitoneal injection and digestive tract absorption. That is, mice were given intraperitoneal injection of DM-AKG100 mg/kg/day 2 h before cisplatin injection and 2, 22, and 46 h after cisplatin injection, respectively. And, during the whole treatment period, mice were provided with drinking water supplemented with 4% DM-AKG. All mice were euthanized at 48 h after cisplatin injection, and blood and kidneys were collected for analysis.

**Fig. 1:**
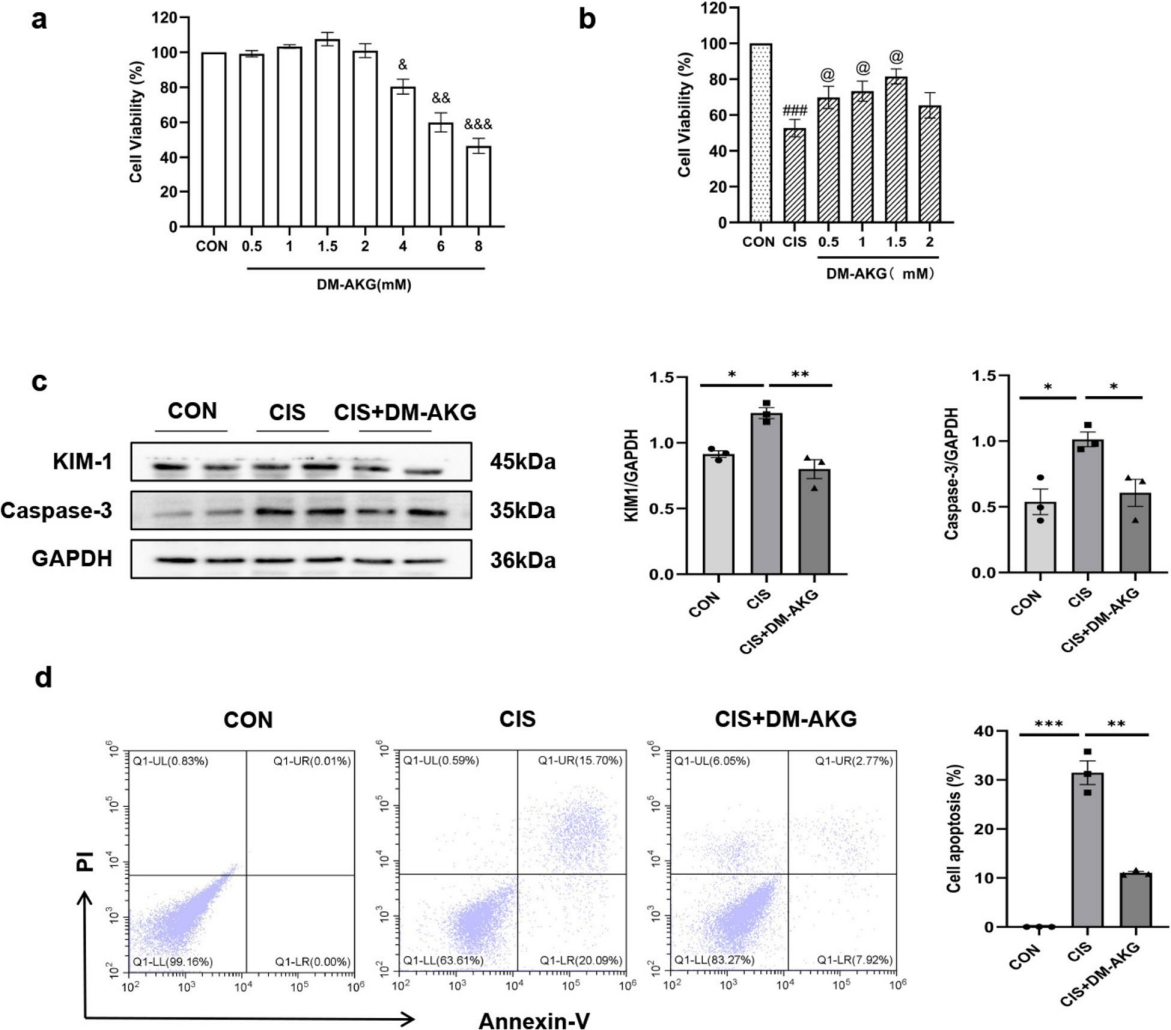
DM-AKG improves the survival rate and reduces apoptosis of HK-2 cells treated with CIS. **a** Different concentrations of DM-AKG were co-incubated with HK-2 cells, and cell viability was detected by CCK-8 after 24 h. Data are presented as mean ± SEM. Compared with the control group, ^&^*p* < 0.05, ^&&^*p* < 0.01, ^&&&^*p* < 0.001. **b** HK-2 cells were pretreated with or without DM-AKG for 4 h and then treated with CIS. CCK-8 evaluated the cell survival rate after 24 h of CIS treatment. Data are presented as mean ± SEM. Compared with control group, ^###^*p* < 0.001. Compared with CIS group, ^@^*p* < 0.05. **c** Protein levels of KIM-1 and Caspase-3 were measured by Western blotting, and the data are expressed as optical density normalized to GAPDH protein. **d** Flow cytometry was used to detect apoptosis, with data expressed as mean ± SEM. ^*^p < 0.05, ^**^p < 0.01, and ^***^p < 0.001

### Measurement of serum creatinine and urea nitrogen

Serum creatinine (Scr) and blood urea nitrogen (BUN) levels were quantified using absorption spectroscopy (Chemray 800, Shenzhen, China) in accordance with the manufacturer’s instructions provided by Servicebio Technology (Wuhan, China).

### Western blot analysis

Mouse kidney tissue and HK-2 cell proteins were extracted using a mixture of RIPA buffer (Beyotime, Jiangsu, China), protease inhibitor (Targetmol, USA), and phosphatase inhibitor (Solarbio, Beijing, China). Protein quantification was performed using the BCA method (Beyotime, Jiangsu, China). The proteins were separated by 10% SDS-PAGE gel electrophoresis and transferred to polyvinylidene difluoride (PVDF) membranes. The membranes were blocked with a protein-free rapid blocking solution and incubated with primary antibodies overnight at 4 °C on a shaker. Subsequently, the membranes were incubated with horseradish peroxidase-conjugated secondary antibodies for 1 h at room temperature. Protein bands were visualized using an extra-ultrasensitive ECL chemiluminescence kit (Beyotime, Jiangsu, China) and a chemiluminescence gel imager (Chemidoc XRS + Bio-Rad, USA). The images were quantified using ImageJ software (NIH, Bethesda, MD, USA).

### CCK-8 assay

Cell suspensions were seeded into 96-well plates at a density of 1 × 10^4^ cells per well and incubated in a 37 °C, 5% CO_2_ environment. After 24 h of cisplatin (CIS) treatment, cell viability was assessed using the Cell Counting Kit-8 (CCK-8) assay (Beyotime, Jiangsu, China) following the manufacturer’s instructions.

### Flow cytometry

Apoptosis was evaluated using an Annexin V-FITC/Propidium Iodide (PI) apoptosis detection kit (Keygentec, Jiangsu, China). Drug-treated HK-2 cells were harvested after digestion with EDTA-free trypsin. The harvested cells were resuspended in 400 μL of binding buffer, incubated with 5 μL of Annexin V-FITC and 5 μL of PI in the dark for 10 min, and then analyzed using a flow cytometer (Beckman Coulter, USA).

### ROS measurements

Intracellular reactive oxygen species (ROS) production was quantified using a commercial kit (DCFH-DA, Beyotime, Jiangsu, China). Cells were incubated with medium containing the fluorescent probe DCFH-DA for 30 min at 37 °C, following the manufacturer’s instructions. After incubation, cells were washed three times with PBS in the dark. Cell images were captured using a laser confocal microscope (Leica TCS SP8, Germany), and the fluorescence density was quantified using ImageJ software.

### Measurement of mitochondrial membrane potential (MMP)

Mitochondrial membrane potential (MMP) changes were assessed using the JC-1 kit (Solarbio, Beijing, China). HK-2 cells were incubated with the JC-1 staining solution for 20 min at 37 °C in a cell culture incubator, following the manufacturer’s instructions. Observations were made using a fluorescence inverted microscope (ZEISS, China). Red fluorescence indicates the presence of J-aggregates (high MMP), while green fluorescence indicates J-monomers (low MMP). The fluorescence density was quantified using ImageJ software.

### Histological analysis

Fresh renal tissues were fixed in 4% paraformaldehyde, then dehydrated through a graded series of alcohols (100%, 95%, 85%, and 75%), and embedded in paraffin. Tissue sections of 5 μm were stained with hematoxylin–eosin (H&E). Renal tubular injury was scored based on the degree of brush border loss, tubular dilation, tubular cast formation, tubular necrosis, and neutrophil infiltration (0: normal; 1: mild injury with 0%–10% involvement; 2: moderate injury with 11%–25% involvement; 3: severe injury with 26%–49% involvement; 4: highly severe injury with 50%–75% involvement; 5: extensive injury with > 75% involvement) [17]. All assessments were performed by two investigators who were blinded to the experimental conditions.

### Immunohistochemistry

Paraffin-embedded kidney Sects. (5 μm) were deparaffinized and subjected to antigen retrieval using ethylenediaminetetraacetic acid (EDTA). The sections were incubated with primary antibodies against PINK1 (1:100) and Parkin (1:100) overnight at 4 °C. Subsequently, the sections were incubated with species-specific secondary antibodies conjugated to horseradish peroxidase (HRP) for 50 min. Images were captured using light microscopy (Nikon Eclipse E100).

### Transmission electron microscopy (TEM)

Kidney tissues were excised into cubes of approximately 1 mm^3^ and fixed in 2.5% glutaraldehyde for 2 h. Subsequently, the tissue fragments were post-fixed in 1% osmium tetroxide for 2 h. Following ethanol dehydration and embedding in resin, ultrathin sections of 60 nm were prepared. These sections were then stained with 2% uranyl acetate for 8 min. The mitochondrial ultrastructure was examined using a transmission electron microscope (Hitachi HT7800/HT7700).

### Network pharmacology

#### Screening of potential AKG targets

We obtained the chemical structural formula and SMILES number of DM-AKG through PubChem (https://pubchem.ncbi.nlm.nih.gov). Potential drug targets for DM-AKG were collected from the SwissTargetPrediction database (http://swisstargetprediction.ch), the Comparative Toxicogenomics Database (CTD) (https://ctdbase.org), and TargetNet (http://targetnet.scbdd.com).

### AKI-related targets

Disease-related targets were obtained from the GeneCards database (https://www.genecards.org/) and the OMIM database (http://omim.org) using the keyword “acute kidney injury”. Subsequently, AKI-related targets were identified by combining the results from these databases and removing duplicates.

### Screening of common targets of AKG and AKI

Common targets of AKG and AKI were identified using the VENNY 2.1 online platform (https://bioinfogp.cnb.csic.es/tools/venny).

### Protein–protein interaction (PPI) network construction

The PPI network of common targets was constructed using the STRING database (https://string-db.org/). The organism was set as Homo sapiens, the confidence score was set to greater than 0.4, and other parameters were kept at default values. Subsequently, the PPI networks were exported in TSV format and analyzed using Cytoscape 3.10.0 (https://cytoscape.org/). The PPI networks were visualized with nodes representing target proteins and edges representing PPIs. The degree represents the number of nodes directly connected to a given node. Core targets were identified using the Cytoscape plug-in for network analysis.

### Gene function and pathway enrichment analysis

Gene Ontology (GO) functional enrichment analysis and Kyoto Encyclopedia of Genes and Genomes (KEGG) pathway enrichment analysis were performed on the core targets using DAVID 6.8 (https://david.ncifcrf.gov/). The top 20 biological processes (BP), cellular components (CC), molecular functions (MF), and KEGG pathways with *p*-values less than 0.05 were selected, excluding disease-related terms such as those related to tumors and infectious diseases.

### Statistical analysis

Data are presented as the mean ± standard error of the mean (SEM). For normally distributed data, comparisons between two groups were made using two-tailed unpaired Student’s t-tests, and comparisons among multiple groups were analyzed by one-way analysis of variance (ANOVA) followed by Tukey’s post hoc test.

All statistical analyses were conducted using GraphPad Prism 10 software (San Diego, CA, USA). A *p*-value of less than 0.05 was considered to indicate statistical significance.

## Results

### DM-AKG ameliorated CIS-treated HK-2 cell injury and reduced apoptosis in vitro

We initially assessed the cytotoxicity of DM-AKG on HK-2 cells using the CCK-8 assay. The results indicated that HK-2 cell viability decreased in a dose-dependent manner when the concentration of DM-AKG exceeded 2 mM. The CC_50_ of DM-AKG for HK-2 cells was determined to be 7.274 mM (Fig. 1a). Subsequently, we established an in vitro model of CIS-AKI by treating HK-2 cells with CIS to evaluate the effects of various concentrations of DM-AKG on cell survival. The findings revealed that 1.5 mM DM-AKG provided optimal protection against CIS-treated HK-2 cell injury, with an EC_50_ of 2.955 mM (Fig. 1b). Furthermore, Western blot analysis demonstrated that DM-AKG treatment significantly reduced the expression of KIM-1 and Caspase-3 in the cells compared to CIS-treated HK-2 cells (all* p* < 0.05) (Fig. 1c). Consistent with these observations, flow cytometry results showed that DM-AKG decreased the percentage of CIS-induced apoptosis in HK-2 cells by 20.42% (*p* < 0.01) (Fig. 1d). These results suggest that DM-AKG mitigates CIS-induced injury to renal tubular epithelial cells in vitro.

### DM-AKG improved renal function and pathological injury in CIS-AKI mice

**Fig. 2:**
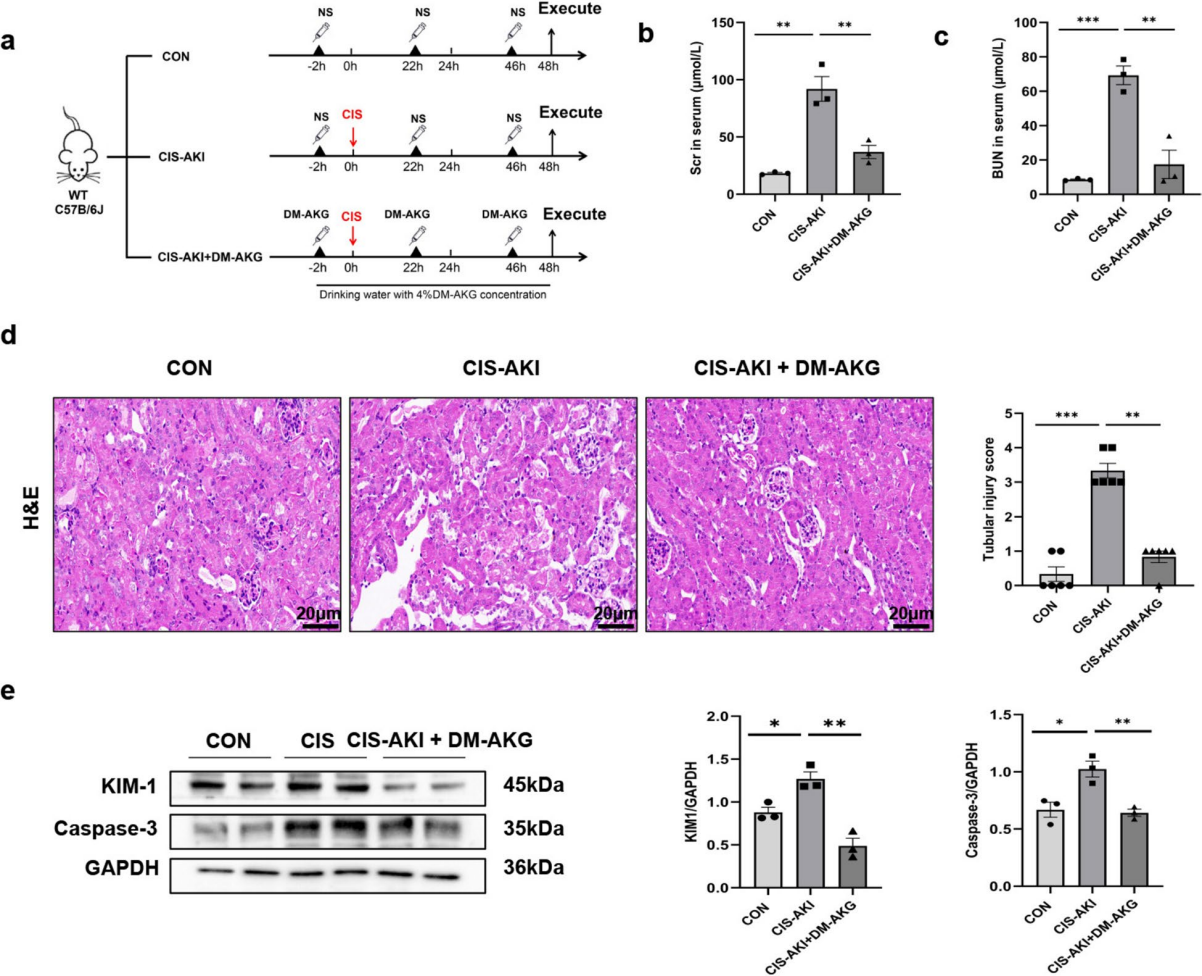
DM-AKG ameliorates CIS-AKI in *vivo*. **a** Schematic diagram of the establishment of the CIS-induced AKI mouse model and DM-AKG treatment. NS indicates saline. **b**, **c** Scr and BUN levels in mouse serum. **d** H&E staining to detect histologic changes in mouse kidney. Scale bar: 20 μm. **e** Western blot was performed to detect KIM-1 and Caspase-3, and the data are expressed as optical densities normalized to GAPDH proteins. The above data are expressed as mean ± SEM (*n* = 6). ^*^p < 0.05, ^**^p < 0.01, and ^***^p < 0.001

As depicted in Fig. 2a, we evaluated therapeutic effect of DM-AKG on CIS-AKI mice. Initially, we assessed the changes in serum markers of AKI. The results indicated that both serum creatinine (Scr) and blood urea nitrogen (BUN) levels were significantly elevated in the CIS-AKI group compared to the control group (*p* < 0.01). Conversely, these levels were significantly reduced in the CIS-AKI + DM-AKG group compared to the CIS-AKI group (*p* < 0.01) (Fig. 2b–c). Subsequently, we examined the expression of the KIM-1 using Western Blot. The findings similarly demonstrated that DM-AKG could reduce KIM-1 expression in the kidney tissues of CIS-AKI mice (*p* < 0.01) (Fig. 2e). Additionally, in the H&E staining assay, the renal cortex of CIS-AKI mice exhibited pronounced pathological changes, including extensive edema and necrosis of renal proximal tubular epithelial cells, detachment of the tubular brush border, partial glomerular atrophy, and the presence of proteinaceous casts and inflammatory cell infiltration. However, DM-AKG treatment significantly ameliorated these pathological changes and reduced the AKI pathological score (*p* < 0.01) (Fig. 2d). Furthermore, we assessed the expression of Caspase-3 protein in renal tissues. As shown in Fig. 2e, compared with CIS-AKI group, the expression of Caspase-3 in CIS-AKI + DM-AKG group decreased significantly (*p* < 0.01). These findings indicate that DM-AKG has therapeutic effect on AKI, by alleviating kidney injury caused by CIS.

### PPI prediction of core targets of AKG for AKI treatment

**Fig. 3:**
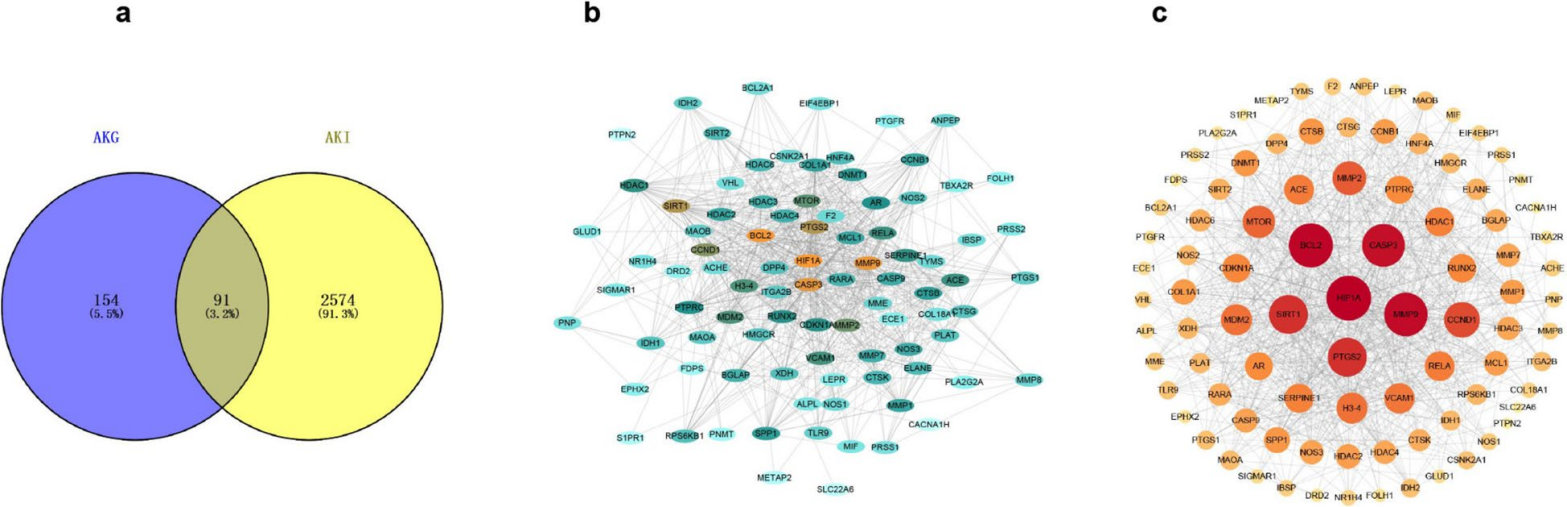
AKG and AKI common targets and PPI network. **a** Venn diagram of overlapping targets between AKG and AKI (note: purple circles indicate genes relevant to the drug; yellow circles indicate genes relevant to the disease). **b**, **c** PPI network diagram for AKG in AKI. Nodes represent target proteins; edges indicate interactions between targets. Node size and color represent degree values; larger nodes and darker colors indicate higher degree values

We further predicted the molecular mechanisms by which AKG ameliorates AKI through network pharmacology. A total of 262 targets associated with AKG utility were identified in the SwissTarget Prediction database, TargetNet database, and Therapeutic Target Database. Additionally, 2665 AKI disease-related targets were obtained from GeneCards and the Online Mendelian Inheritance in Man (OMIM) database. After intersecting these datasets using a Venn diagram, 91 potential targets of action for AKG in AKI were identified (Fig. 3a). We uploaded these 91 action targets into the STRING database and constructed a protein interaction network using an interaction score greater than 0.400, while hiding the free nodes. The resulting network graph contained 90 nodes and 783 edges, describing the interactions between the targets (Fig. 3b). The protein interaction networks in the STRING database were then ranked using the Cytoscape median value algorithm (Fig. 3c) [18]. In these PPI networks, proteins such as HIF1A, PTGS2, SIRT1, MMP9, CASP3, BCL2, CCND1, ACE, MTOR, and HDAC6 show more intensive interaction. Among them, HIF1A, SIRT1, MTOR, and HDAC6 are closely related to cell energy metabolism or mitochondrial function [7, 19–22].

### GO and KEGG prediction of key pathways for AKG therapy for AKI

**Fig. 4:**
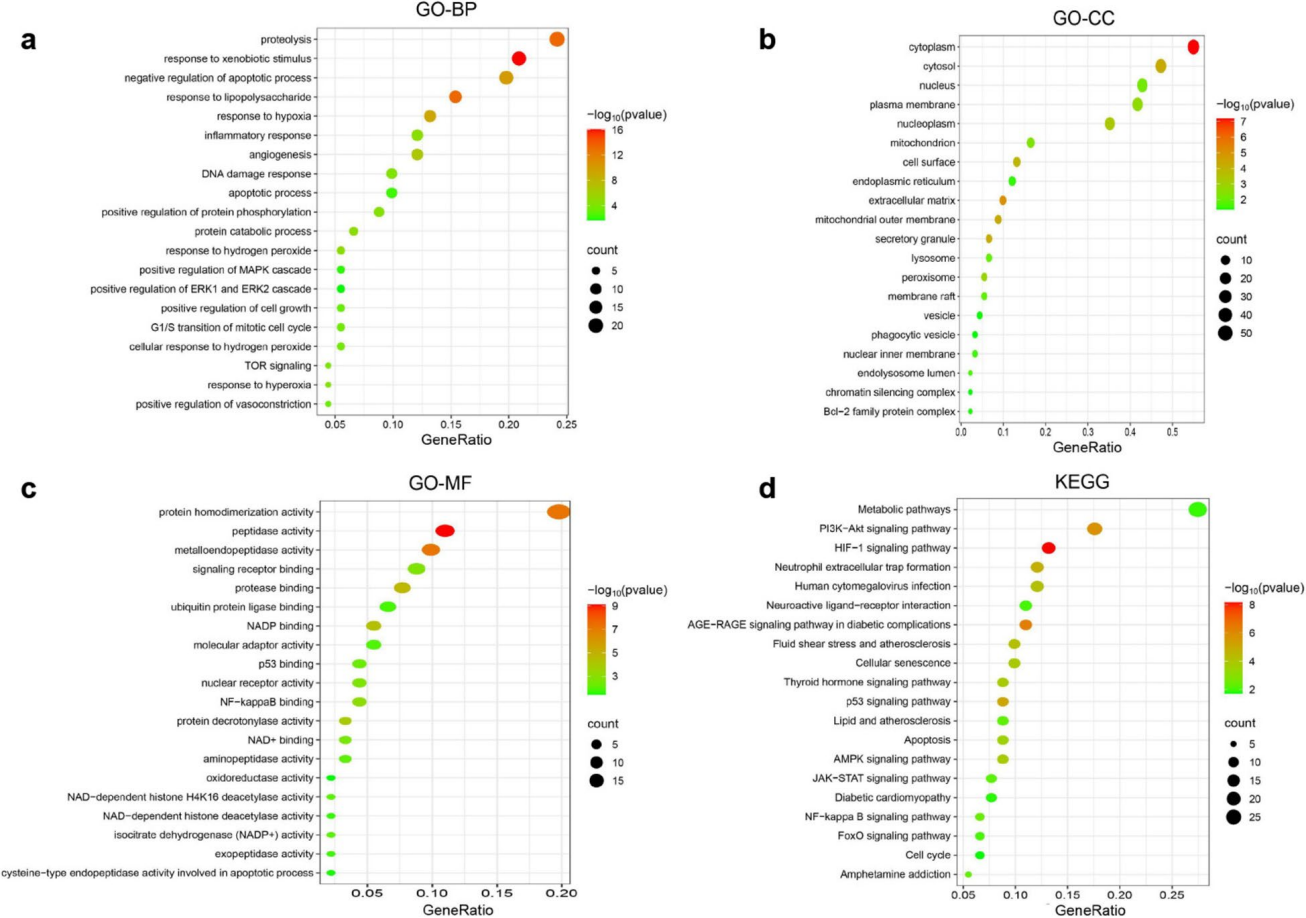
GO enrichment analysis and KEGG pathway enrichment analysis. **a**–**c** Bubble plots showing the top 20 major biological processes (BPs), cellular components (CCs), and molecular functions (MFs) generated by GO enrichment analysis. The X-axis shows gene ratios, colors indicate *p*-values, and bubble sizes indicate counts of term-enriched targets. **d** Bubble plots showing the top 20 major KEGG pathways. The X-axis shows gene ratios, color indicates *p*-value, and bubble size indicates counts of term-enriched targets

GO and KEGG are powerful tools for pathway enrichment and analysis within interaction networks, allowing us to explore the biological processes and pathways related to drug action. Therefore, we next conducted GO enrichment analysis of the 91 intersecting gene action targets using the DAVID 6.8 database. The analysis yielded 73 GO-related entries. Among these, 264 biological processes (BPs) were primarily involved in responses to hydrogen peroxide, protein phosphorylation, and energy metabolism (Fig. 4a). Additionally, 42 cellular components (CCs) were identified, with a focus on mitochondria, lysosomes, peroxisomes, and phagocytic vesicles, among others (Fig. 4b). Furthermore, 73 molecular functions (MFs) were noted, mainly involving isocitrate dehydrogenase (NADP +) activity, oxidoreductase activity, and ubiquitin-like protein ligase binding (Fig. 4c). We performed KEGG pathway enrichment analysis using the DAVID 6.8 database, which resulted in 77 entries. As illustrated in Fig. 4d, metabolism-related pathways were most enriched in the KEGG results. Notably, signaling pathways such as PI3K-Akt, AMPK, HIF-1, AGE-RAGE, and FoxO were prominently featured.

### DM-AKG reduces oxidative stress and improves mitochondrial membrane potential and mitochondrial kinetics in CIS-treated HK-2 cells

**Fig. 5:**
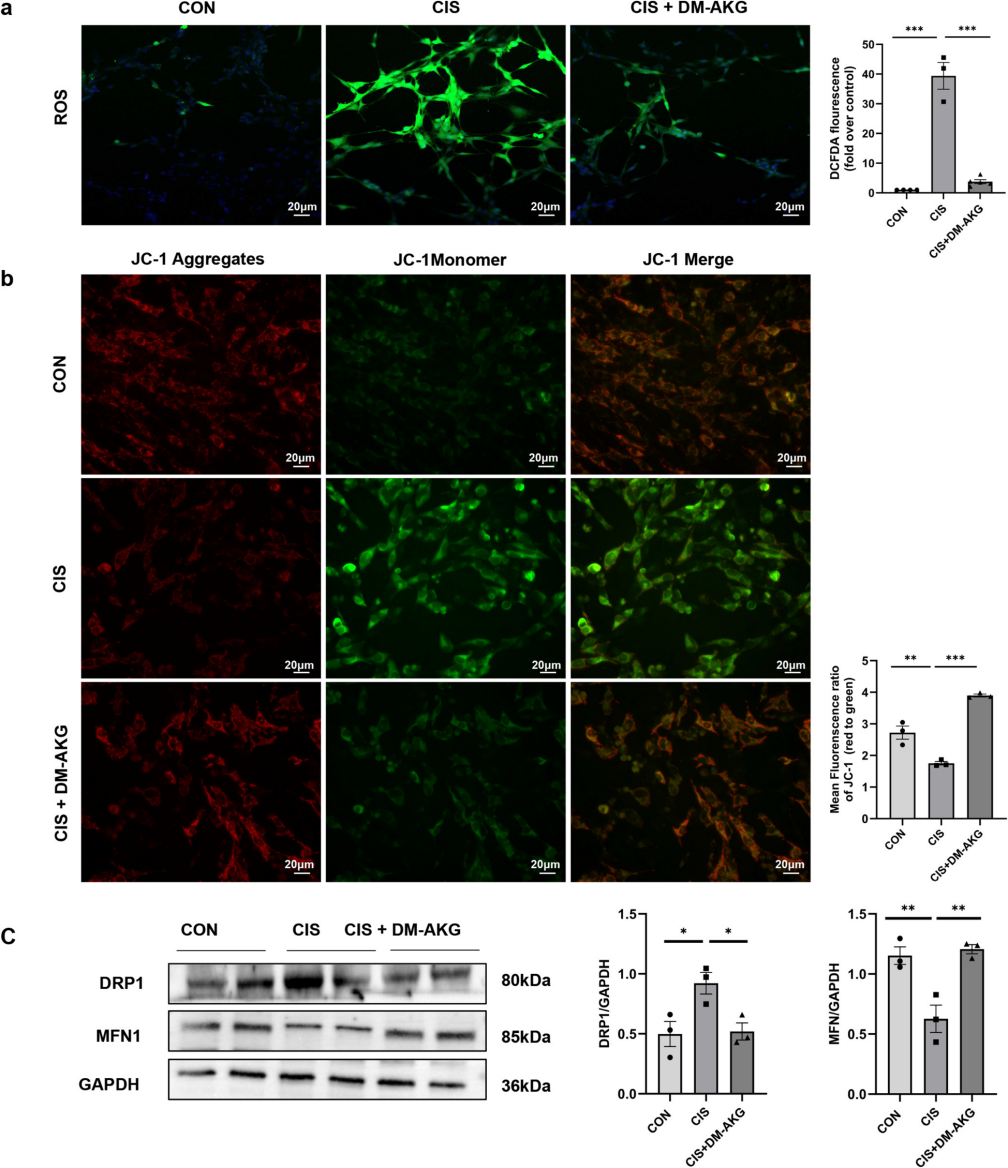
Effects of DM-AKG on ROS, MMP, and mitochondrial dynamics in vitro. **a** DCFH-DA staining (green) was used to detect the level of ROS in cells. **b** JC-1 staining was used to detect MMP level in cells. **c** Western blot analysis to determine the expression of DRP1 and MFN1 proteins. Data are expressed as optical density normalized to GAPDH protein. Values represent mean ± SEM. ^*^p < 0.05, ^**^p < 0.01, and ^***^p < 0.001

Based on the network pharmacology analysis results, we further investigated the effects of DM-AKG on mitochondrial function and dynamics in HK-2 cells treated with CIS. Initially, we evaluated the mitochondrial functional status by examining the changes in reactive oxygen species (ROS) and mitochondrial membrane potential (MMP) within the cells. The results indicated a significant increase in ROS fluorescence (*p* < 0.001) and a significant decrease in MMP green fluorescence intensity (*p* < 0.01) in the CIS group compared to the control group (Fig. 5a–b). However, ROS production was significantly reduced and MMP was elevated in the CIS + DM-AKG group compared to the CIS group (*p* < 0.001) (Fig. 5a–b). These findings suggest that DM-AKG supplementation mitigates CIS-induced oxidative stress injury and mitochondrial dysfunction. Subsequently, we assessed mitochondrial kinetic function by examining the expression of MFN1 and DRP1. The results showed that MFN1 expression was decreased (*p* < 0.01) and DRP1 expression was increased (*p* < 0.05) in CIS-treated HK-2 cells compared to the control group, indicating that mitochondria exhibited kinetic alterations characterized by decreased fusion and increased fission in CIS-AKI (Fig. 5c). In contrast, MFN1 protein expression was increased (*p* < 0.01) and DRP1 protein expression was decreased (*p* < 0.05) following DM-AKG supplementation compared to the CIS group. In conclusion, these results suggest that DM-AKG effectively ameliorates CIS-induced mitochondrial dysfunction in HK-2 cells.

### DM-AKG improves mitochondrial function in CIS-AKI mice

**Fig. 6:**
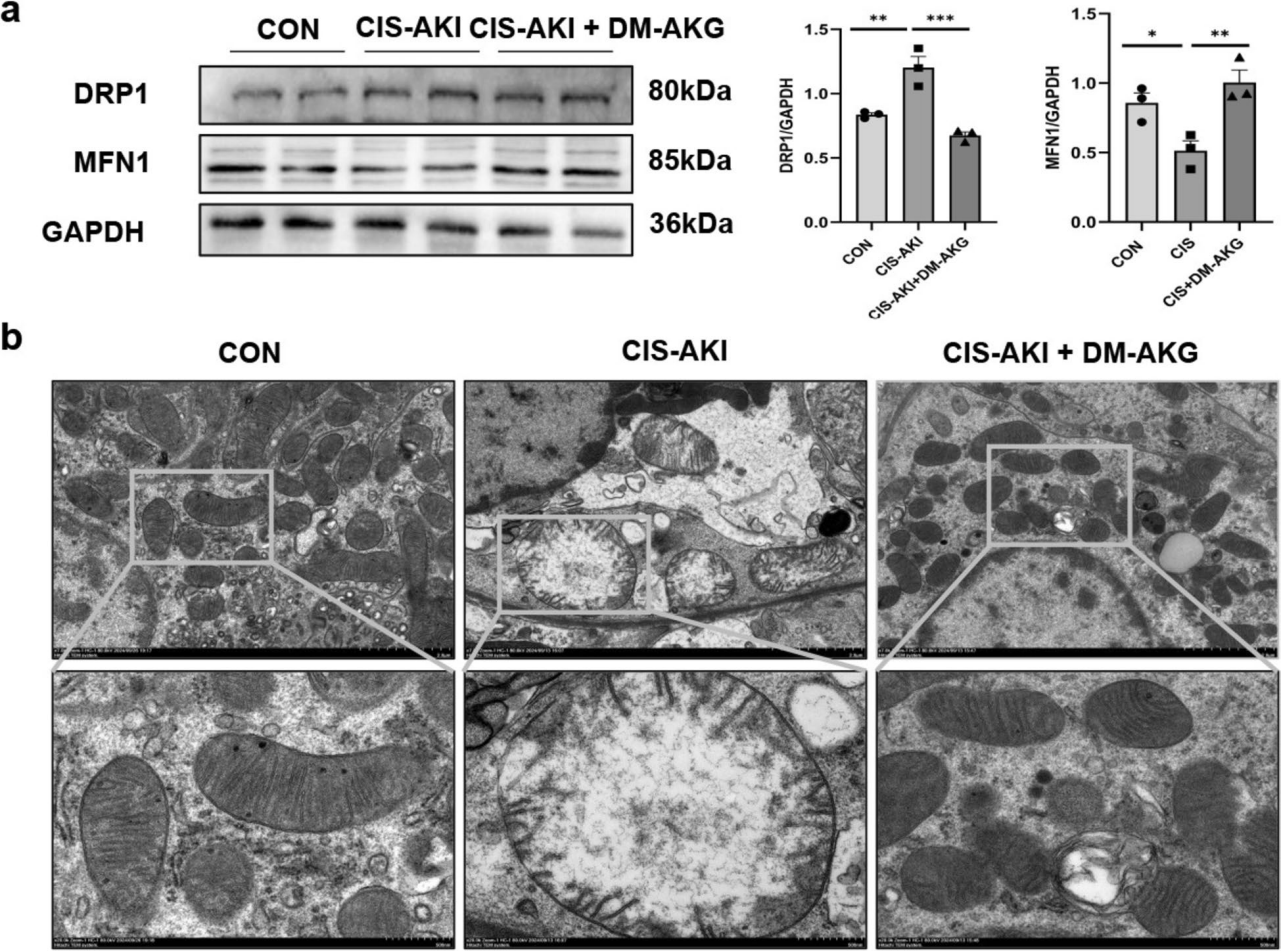
Effects of DM-AKG on mitochondrial dynamics and mitochondrial structure of renal tubular cells in vivo. **a** Western blot analysis of DRP1 and MFN1 proteins in mouse kidney tissues. Data are expressed as optical density normalized to GAPDH protein. **b** Morphological changes of mitochondria were detected by TEM. Scale bar: 2.0 μm, 500 nm. ^*^p < 0.05, ^**^p < 0.01, and ^***^p < 0.001

In addition, we evaluated the effect of DM-AKG on mitochondrial function in kidney tissue of CIS-AKI mice. Immunoblotting results consistently demonstrated that DM-AKG treatment significantly prevented the reduction of MFN1 (*p* < 0.01) and the increase of DRP1 expression (*p* < 0.001) in the kidney tissues of CIS-AKI mice (Fig. 6a). Correspondingly, electron microscopy results revealed an increase in the number of mitochondria, a normalization of mitochondrial morphology, and an enhancement of mitochondrial cristae in the renal tissues of DM-AKG-treated CIS-AKI mice (Fig. 6b). These findings confirm that DM-AKG significantly improved the mitochondrial dysfunction in kidney of CIS-AKI mice.

### DM-AKG promotes mitophagy in CIS-AKI mice

**Fig. 7:**
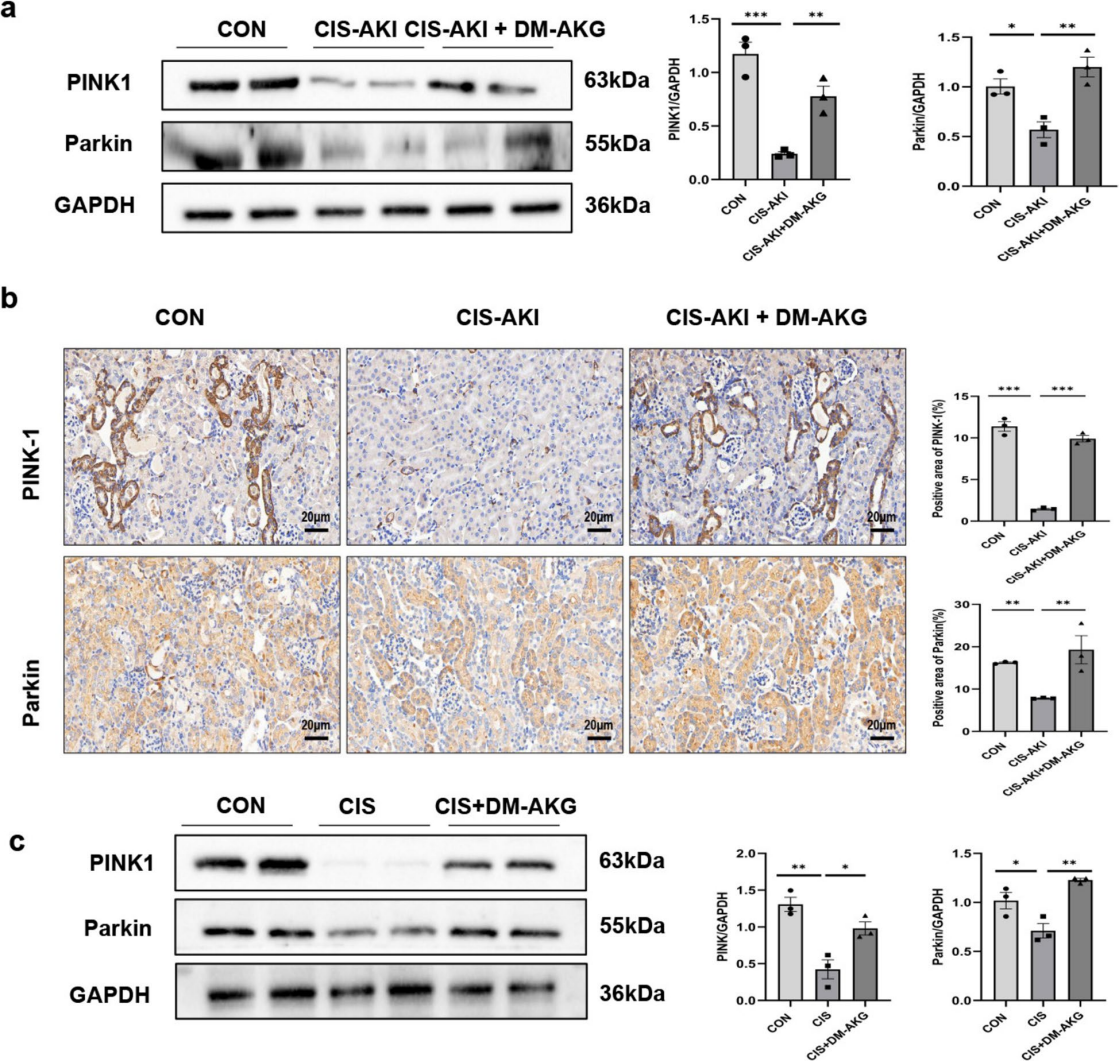
Effect of DM-AKG on the mitophagy pathway in vivo and vitro. **a** Western blot analysis of PINK1 and Parkin proteins in mouse kidney tissues. **b** Detection of PINK1 and Parkin expression using immunohistochemical staining. Scale bar: 20 μm. **c** Western blot determination of PINK1 and Parkin proteins in HK-2 cells. Data are expressed as optical density normalized to GAPDH protein. All data are expressed as mean ± SEM (*n* = 6 mice/group). ^*^p < 0.05, ^**^p < 0.01, and ^***^p < 0.001

The initiation and execution of mitophagy are mediated by a variety of signaling pathways and molecular interaction networks, with the PINK1/Parkin pathway being one of the most intensively studied regulatory mechanisms. Consequently, to investigate whether the improvement of mitochondrial function in CIS-AKI by DM-AKG is associated with the regulation of mitochondrial autophagy, we examined the expression levels of proteins related to the PINK1/Parkin pathway. In vivo experiments, both Western blot and immunohistochemistry analyses showed that the expression of PINK1 and Parkin in the kidney tissues of mice in the CIS-AKI group was lower than that in the control group (*p* < 0.05), and this reduction was significantly reversed (*p* < 0.01) following DM-AKG treatment (Fig. 7a–b). Consistent with these findings, our in vitro experiments revealed that the expression of PINK1 and Parkin in CIS + DM-AKG group was significantly higher than that in the HK-2 cells treated with CIS alone (*p* < 0.05) (Fig. 7c). Importantly, electron microscopy assays of kidney tissues from DM-AKG-treated CIS-AKI mice showed a significant increase in mitophagy (Fig. 6b). Collectively, these results suggest that DM-AKG promotes mitophagy via the PINK1/Parkin pathway, thereby improving mitochondrial function in CIS-AKI.

## Discussion

In this study, we explored the therapeutic potential of DM-AKG in CIS-AKI and elucidated its underlying molecular mechanisms. Our results indicate that DM-AKG confers significant therapeutic advantages in a CIS-AKI model, effectively enhancing renal function and mitigating histopathological kidney damage. Moreover, we discovered that the therapeutic efficacy of DM-AKG is intimately linked to its capacity to modulate energy metabolism and restore mitochondrial function. On the molecular mechanism, we demonstrated that DM-AKG stimulates mitophagy by activating the PINK1/Parkin signaling pathway, thereby enhancing mitochondrial function. This discovery not only furnishes novel molecular insights into the nephroprotective role of DM-AKG but also establishes a crucial theoretical foundation for the development of novel therapeutic strategies against diseases associated with mitochondrial dysfunction.

In previous studies, Anja Bienholz et al. recognized the potential value of AKG in disease treatment and explored its efficacy in ischemia/reperfusion (I/R)-induced AKI [23]. They found that the combination of supplemental α-ketoglutarate and malic acid (AKG/MAL) had a significant protective effect against hypoxia-induced injury in isolated proximal tubules [24, 25]. However, in the I/R-induced acute kidney injury model of male Sprague–Dawley rats, the expected improvement effect was not observed by continuous infusion of AKG/MAL 60 min before ischemia and AKG/MAL 1 mmol/kg 45 min after ischemia. This may be attributed to the poorer cell membrane penetration of DM-AKG, which limits the bioavailability of exogenous AKG in vivo [26]. However, in the present study, we found that DM-AKG showed a significant improvement in the progress of CIS-induced-AKI in cell and mouse models. This difference in results may be related to the following factors: first, the main sites of action of DM-AKG are in the cytoplasm and mitochondria, whereas DM-AKG may have better cell membrane penetration due to its modified chemical structure, which improves its bioavailability in vivo [27]. Secondly, regarding the drug delivery strategy, we employed a combination of intraperitoneal injection and administration via drinking water. This approach was designed to maintain a more sustained drug concentration in the body. What can support our improved medication strategy is that exogenous AKG is well absorbed and metabolized in the stomach and duodenum, but it is quickly eliminated in the blood [28, 29]. Thus, our results suggest that DM-AKG is a very promising drug for improving the progression of CIS-AKI, but more attention needs to be given to the dosing strategy, i.e., a single dose may not satisfy the pharmacokinetic requirements for disease treatment. These findings highlight the importance of optimization in drug design and dosing strategy to achieve the maximum potential of DM-AKG in CIS-AKI therapy.

Previous studies have demonstrated that AKG, as a pivotal intermediate in the tricarboxylic acid cycle, plays a crucial role in cellular processes such as energy metabolism, amino acid/protein synthesis, and epigenetic regulation [30]. This suggests that AKG functions as a multi-target, multi-pathway, and multi-functional agent. Consequently, we employed network pharmacology—a method capable of integrating large volumes of complex data to elucidate the systematic mechanisms of drug action rather than focusing solely on a single target—to analyze the mechanism of action of AKG. Our results revealed that protein–protein interaction (PPI) data clearly indicate the presence of 91 proteins with frequent interactions between AKG and AKI. Further pathway enrichment analysis showed that AKG can influence AKI’s energy metabolism through multiple pathways, including oxidative stress and reactive oxygen species, as well as via involvement in cellular components such as mitochondria, peroxidases, and Bcl-2 family protein complexes. Additionally, AKG modulates signaling molecules such as AMPK, PI3K-Akt, NF-κB, and FoxO. For instance, during AKI, there is impairment of the tricarboxylic acid cycle and oxidative phosphorylation, an enhancement of glycolysis, a reduction in fatty acid β-oxidation, and persistent disturbances in amino acid metabolism [31]. These metabolic alterations may further exacerbate pathological processes such as oxidative stress, apoptosis, inflammation, and fibrosis in renal tubular cells, leading to aggravated renal injury or maladaptive repair [32, 33]. Additionally, ATX-304, a pan-AMPK activator, has been shown to protect against cisplatin-mediated injury by altering metabolic reprogramming, including fatty acids, tricarboxylic acid cycle metabolites, and amino acids [34]. The flavonoid fisetin attenuates renal inflammation and apoptosis in septic AKI mice through NF-κB and MAPK signaling pathways [35]. MiR-21-3p mediates energy metabolism and apoptosis in tubular epithelial cells (TEC) by manipulating the AKT/CDK2-FOXO1 pathway, thereby ameliorating sepsis-associated acute kidney injury (SAKI) progression [36]. Therefore, this information suggests that DM-AKG may ameliorate CIS-AKI by regulating cellular energy metabolism.

Mitochondrial damage and dysfunction are considered to play a key role in exploring the core mechanism of TEC energy metabolism imbalance [37]. It is generally accepted that mitochondria, as the main site of key energy metabolic pathways such as the tricarboxylic acid cycle, oxidative phosphorylation, fatty acid β-oxidation, and ketone body oxidation, constitute the core network of cellular energy metabolism and provide the cell with essential energy and intermediate metabolites [38]. Therefore, maintaining mitochondrial homeostasis is critical for preventing the onset and delaying the progression of kidney diseases [3, 39]. Notably, in both in vitro and in vivo experiments, we found that DM-AKG treatment significantly increased the expression level of MFN1 and decreased the expression level of DRP1. This finding suggests that DM-AKG may act by regulating mitochondrial dynamics in renal tubular epithelial cells. Mitochondrial dynamics refers to the dynamic regulation of the number and function of mitochondria through a continuous process of fusion and division, a process that involves changes in mitochondrial biosynthesis, MMP, and ROS content, and is critical for the maintenance of mitochondrial morphology, number, and function. This was supported by further experimental results, in which DM-AKG significantly reduced ROS content and MMP levels in CIS-treated HK-2 cells. In addition, in the kidneys of DM-AKG-treated CIS-AKI mice, we observed an increase in the number of mitochondria, a restoration of mitochondrial morphology, and an increase in mitochondrial cristae, which are results that further confirm the positive effects of DM-AKG on mitochondrial homeostasis.

Mitophagy is a selective autophagic process that targets damaged or excess mitochondria for degradation via double-membrane vesicle encapsulation, playing an indispensable role in maintaining intracellular mitochondrial quality control and overall normal function [40]. Studies have shown that in cells and mice with impaired mitophagy due to PINK1 or PARK2 defects, renal injuries such as mitochondrial damage, mitochondrial ROS, and apoptosis are more severe under contrast or LPS exposure than in the wild-type group [39, 41]. However, overexpression of PINK1/Parkin has been shown to protect against cisplatin-induced mitochondrial dysfunction and cellular damage by promoting mitophagy [42]. In our study, we observed that DM-AKG supplementation increased the expression of PINK1/Parkin in CIS-AKI mice. This provides critical molecular-level evidence that DM-AKG mediates mitophagy to improve mitochondrial function. Of interest, a study by Hao Yu et al. found that AKG was able to ameliorate pressure overload-induced cardiac insufficiency in mice by promoting mitophagy and regulating ferroptosis [11]. Notably, we also captured an increase in the number of mitochondrial autophagic lysosomes in renal tissues of CIS-AKI mice after supplementation with DM-AKG, which is strong evidence for the occurrence of mitophagy.

In conclusion, our findings reveal that DM-AKG confers significant therapeutic benefits in the CIS-AKI model, effectively improving renal function and reducing histopathological damage in the kidney. Additionally, our study underscores the role of DM-AKG in regulating cellular energy metabolism by enhancing mitochondrial function through PINK1/Parkin-mediated mitophagy. However, there are several limitations to our study. For instance, the role and molecular mechanisms of DM-AKG in AKI are not yet fully elucidated and require further exploration using experimental strategies such as gene editing. Moreover, the effect of DM-AKG on energy metabolites in CIS-AKI needs to be confirmed through metabolomics approaches. Furthermore, the efficacy of DM-AKG under different administration strategies is worth considering. Future research should aim to address these limitations to fully harness the potential of DM-AKG in AKI therapy.

## Data Availability

The data supporting the findings of this study are available within the article.
